# Feasibility of electroacupuncture at Baihui (GV20) and Zusanli (ST36) on survival with a favorable neurological outcome in patients with postcardiac arrest syndrome after in-hospital cardiac arrest: study protocol for a pilot randomized controlled trial

**DOI:** 10.1186/s40814-023-01239-9

**Published:** 2023-01-13

**Authors:** Ruifeng Zeng, Fang Lai, Manhua Huang, Decai Zhu, Baijian Chen, Lanting Tao, Wei Huang, Chengzhi Lai, Banghan Ding

**Affiliations:** 1 The Second Affiliated Hospital of Guangzhou University of Chinese Medicine, Guangdong Provincial Key Laboratory of Research on Emergency in TCM, Guangzhou, 510120 Guangdong China; 2 Guangdong Provincial Hospital of Chinese Medicine, Guangzhou, 510120 Guangdong China; 3 Fangcun Branch Hospital of Guangdong Provincial Hospital of Chinese Medicine, Guangzhou, 510145 Guangdong China; 4 Ersha Branch Hospital of Guangdong Provincial Hospital of Chinese Medicine, Guangzhou, 510105 Guangdong China

**Keywords:** Cardiac arrest, Postcardiac arrest syndrome, Cardiopulmonary resuscitation protocol, Electroacupuncture

## Abstract

**Background:**

At present, even the first-line medication epinephrine still shows no evidence of a favourable neurological outcome in patients with sudden cardiac arrest (SCA). The high mortality of patients with postcardiac arrest syndrome (PCAS) can be attributed to brain injury, myocardial dysfunction, systemic ischaemia/reperfusion response, and persistent precipitating pathology. Targeted temperature management, the only clinically proven method in the treatment of PCAS, is still associated with a series of problems that have not been completely resolved.

Acupuncture is a crucial therapy in traditional Chinese medicine. On the basis of the results of previous studies, we hypothesize that electroacupuncture (EA) might provide therapeutic benefits in the treatment of PCAS. This study will explore the feasibility of EA on SCA patients.

**Methods:**

This is a prospective pilot, randomized controlled clinical trial. Eligible patients with PCAS after in-hospital cardiac arrest (IHCA) admitted to our department will be randomly allocated to the control group or the EA group. Both groups will receive standard therapy according to American Heart Association guidelines for cardiopulmonary resuscitation. However, the EA group will also receive acupuncture at the Baihui acupoint (GV20) and Zusanli acupoint (ST36) with EA stimulation for 30 min using a dense-dispersed wave at frequencies of 20 and 100 Hz, a current intensity of less than 10 mA, and a pulse width of 0.5 ms. EA treatment will be administered for up to 14 days (until either discharge or death). The primary endpoint is survival with a favourable neurological outcome. The secondary endpoints are neurological scores, cardiac function parameters, and other clinical parameters, including Sequential Organ Failure Assessment (SOFA) scores and Acute Physiology and Chronic Health Evaluation (APACHE) II scores, on days 0 to 28.

**Discussion:**

This study will provide crucial clinical evidence on the efficacy of EA in PCAS when used as an adjunctive treatment with AHA standard therapy.

**Trial registration:**

chictr.org.cn: ChiCTR2000040040. Registered on 19 November 2020. Retrospectively registered. http://www.chictr.org.cn/.

**Supplementary Information:**

The online version contains supplementary material available at 10.1186/s40814-023-01239-9.

## Background

Sudden cardiac arrest (SCA) is a significant cause of death worldwide, and more than 550,000 deaths due to SCA occur annually in the USA and Europe [1]. A recent study showed that epinephrine, the first-line medication for patients with SCA, was associated with more severe neurological impairment in survivors than placebo in adults with out-of-hospital cardiac arrest (OHCA) [2]. In addition, SCA patients will have sepsis-like changes, which may be caused by ischaemia, hypoxia, and reperfusion injury, after the return of spontaneous circulation (ROSC), resulting in multiple organ dysfunction syndromes (MODSs) and a poor prognosis,. This series of reactions is called postcardiac arrest syndrome (PCAS) [3]. According to studies performed in the USA, Europe, and Asia, whether in adults, children, or infants, the mortality rate of PCAS patients is 65–80% [4–8]. The high mortality rate can be attributed to a unique pathophysiological process. The special features of this unique pathophysiological process of PCAS are often superimposed on the disease or injury that caused the cardiac arrest and underlying comorbidities [3]. There are four critical components of PCAS: brain injury [9, 10], myocardial dysfunction [11, 12], systemic ischaemia/reperfusion response [13], and persistent precipitating pathology [14]. Therefore, therapies focusing on a single organ may further damage other damaged organ systems. Targeted temperature management (TTM) is currently the only clinically proven method to improve SCA patients’ survival and neurological outcomes after the ROSC [15, 16]; however, TTM is still associated with a series of problems that have not been completely resolved [17].

Acupuncture is a crucial therapy in traditional Chinese medicine (TCM). Acupuncture involves the insertion of fine needles at “acupoints”, followed by stimulation via manual or mechanical techniques [18]. It has been reported that acupuncture may have a bidirectional regulating effect and an anti-systemic inflammatory response effect [19, 20]. The acupuncture effect of balancing the autonomic nervous system has been demonstrated to inhibit neuronal apoptosis and reduce oxidative stress [21–23]. These effects are in line with the critical components of PCAS mentioned above.

Nevertheless, the effects of acupuncture on PCAS have never been investigated comprehensively. Therefore, the present pilot, randomized, controlled study was designed to explore the feasibility of electroacupuncture (EA) on PCAS patients and furthermore evaluate the impact in the main trial.

The specific objectives of this pilot study were to test the trial procedures, explore the feasibility of the program, and provide data for recruitment, follow-up rates, and sample size calculation for the main trial.

## Method

### Aim

The study is a prospective randomized controlled pilot trial designed to evaluate whether EA can improve survival with a favorable neurological outcome of PCAS after in-hospital cardiac arrest (IHCA).

### Design

This study is a prospective randomized controlled pilot clinical trial that will be carried out in the Emergency Department of Guangdong Provincial Hospital of Chinese Medicine and Intensive Care Unit of Fangcun Branch Hospital of Guangdong Provincial Hospital of Chinese Medicine. This trial is embedded with a study of survival with a favourable neurological outcome of PCAS following IHCA.

The trial received ethics approval from the Guangdong Provincial Hospital of Chinese Medicine Institutional Review Board (approval number ZF2020-051-01). If any changes are made to this protocol, a draft of the new version must be submitted for approval by the Institutional Review Board of Guangdong Provincial Hospital of Chinese Medicine. This clinical trial will conform to the national laws and the principles of the Declaration of Helsinki. The eligible participants’ authorized representatives will sign informed consent papers before the participants are enrolled.

The protocol adheres to the Standard Protocol Items: Recommendations for Interventional Trials (SPIRIT) guidelines (Additional file 1).

### Participant selection criteria

Patients aged 18 to 85 years who suffer SCA in the hospital caused by respiratory failure or hypovolemic shock and with a ROSC sustained for ≥ 20 min and met the following criteria at the same time are eligible to enrol in this study: Glasgow coma score ≤ 8 after ROSC, and before sedation (if any); the legal representative signed the informed consent; advanced cardiovascular life support is conducted. Those who meet the exclusion criteria (listed in Table 1) will not be enrolled in this study.

**Table 1:** Inclusion and exclusion criteria

A. Inclusion criteria for observational part	
1. Cardiac arrest is caused by respiratory failure or hypovolemic shock;	
2. In-hospital cardiac arrest;	
3. Aged 18–85 years old;	
4. Patients with return of spontaneous circulation (ROSC) that is sustained for ≥ 20 min;	
5. Glasgow coma score ≤ 8 after ROSC, and before sedation (if any) ;	
6. The legal representative signed the informed consent;	
7. Advanced cardiovascular life support is conducted.	
B. Exclusion criteria	
1. No-flow time > 10 min (time from collapse to initiation of external cardiac massage);	
2. Low-flow time > 60 min (time from initiation of external cardiac massage to ROSC);	
3. Major hemodynamic instability (defined as a continuous epinephrine or norepinephrine infusion at a flow rate > 1 μg/kg/min);	
4. Cardiac arrest caused by advanced tumor and other end-of-state diseases;	
5. Cardiac arrest caused by an irreversible cause (such as severe trauma and poisoning);	
6. Cardiogenic cardiac arrest (acute infarction, malignant arrhythmia, heart failure, etc.);	
7. Acupoint with lesions, wounds, or skin diseases affects acupuncture;	
8. Allergic persons, or known to be allergic to treatment, such as metal;	
9. Pregnant or lactating women.	

**Fig. 1:**
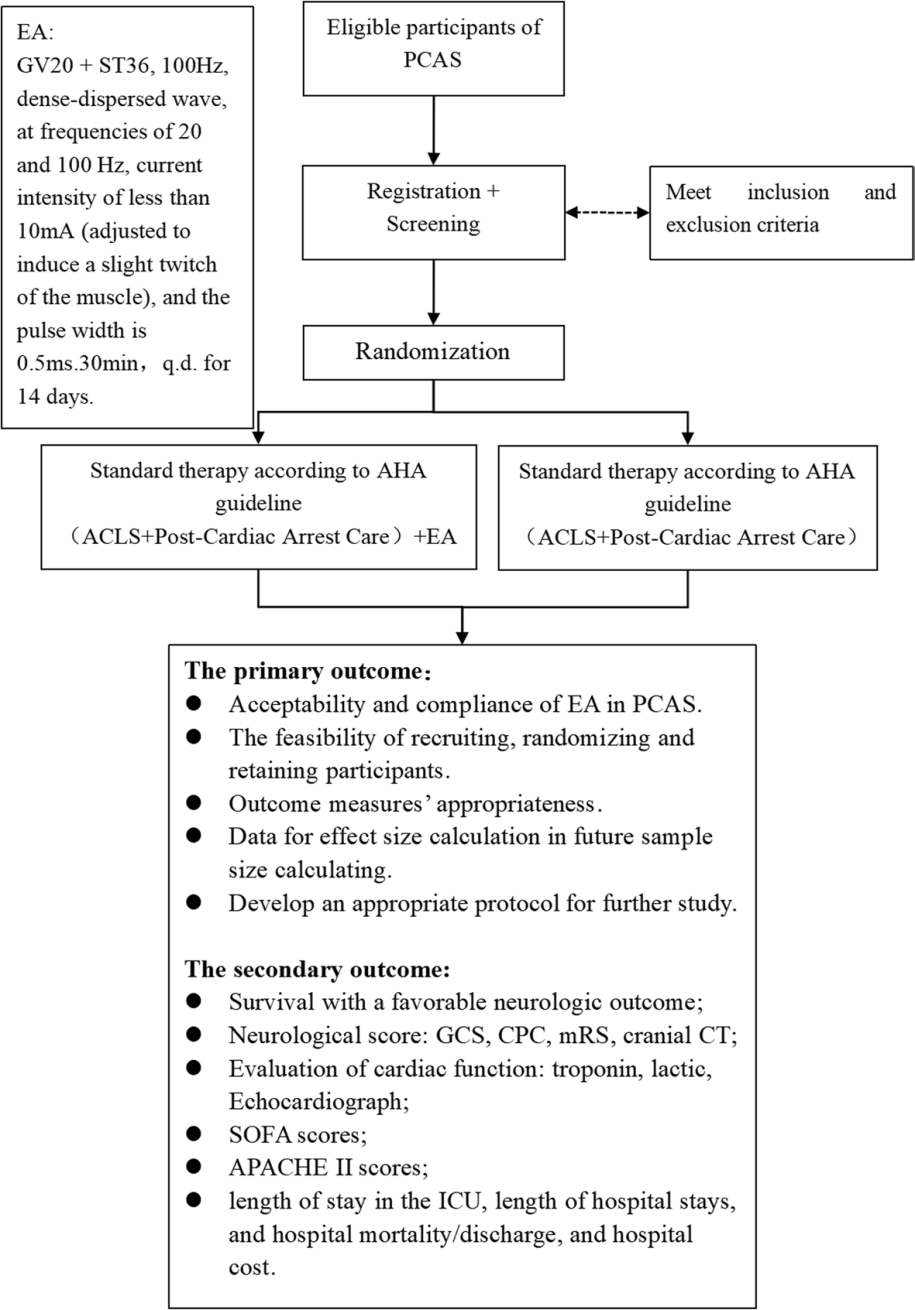
Study flow chart. CA, cardiac arrest; ROSC, return of spontaneous circulation; BLS, basic life support; CPR, cardiopulmonary resuscitation; ACLS, advanced cardiovascular life support; ICU, intensive care unit; EA, electroacupuncture; CPC, cerebral performance category; GCS, Glasgow Coma Scale; CT, computed tomography; SOFA, Sequential Organ Failure Assessment; APACHE, Acute Physiology and Chronic Health Evaluation

Participants can withdraw from the trial for any reason at any time. Participants for whom, upon regaining consciousness (if any), the legal representative or patient requests study withdrawal may be removed from the study:

The flow chart of the study is presented in Fig. 1.

### Randomization

Eligible patients enrolled in this trial will be randomly assigned to the EA or control groups in a 1:1 ratio. Randomization assignment will be carried out by a researcher (Fang Lai) not involved in the treatment and assessment. This randomization will be run with a block randomization size of four by SPSS 17.0. The randomization block size will be concealed to guarantee rigorous methodology, and the allocation number will be sequentially assigned and stored. The unique code assigned to each newly eligible participant will be adequately preserved throughout this study. This procedure will ensure that each eligible patient could be assigned to either of the two groups with equal probability and that group assignment will not be influenced by the researchers. Decai Zhu and Baijian Chen will enrol and assign participants to interventions according to the preprepared allocation sequence. Considering that an included patient could withdraw from the study, their randomization number would be invalidated, so we will ultimately take the first 52–60 patients. Finally, the number of patients in these two groups may be different because of the actual procedures performed.

### Blinding and masking

Since this study involves acupuncture, it is challenging to achieve double blindness. However, to avoid bias in this process, the following specific measures are proposed:

1. Participants will be recruited in strict accordance with the principle of randomization.
2. The physicians participating in the outcome evaluation will be nonresearchers, making the trial efficacy evaluation blinded.
3. The laboratory technicians and the biostatisticians will be blinded to the assigned treatments.

### Sample size determination

Since there is no previous clinical study on EA to improve the survival of patients with PCAS and given this trial's pilot nature, we will follow the recommendation of 20 participants or more to achieve sufficient precision based on the sample size calculation of a previous study [24]. We plan to recruit 50 participants since a 20% dropout rate is expected.

### Interventions and procedures

Eligible patients for whom consent is provided by the legal representative or the patient themselves will receive EA. In addition, participants will receive standard therapy according to American Heart Association guidelines for CPR recommendations [14, 25–27], while any other adjunct treatment will be prohibited. Investigators will be trained before the start of the study. Fang Lai, who will not be involved in participants’ enrolment or data analysis, will monitor the outcomes of the interventions at least once per month during the trial.

### Standard therapy

Standard therapy will be based on current recommendations [14, 25–27], including basic life support (BLS), advanced cardiovascular life support (ACLS), and postcardiac arrest care. BLS includes immediate recognition of SCA, EMS activation, early CPR, and rapid defibrillation. ACLS consists of airway control and ventilation, antiarrhythmic drugs and vasopressors during and immediately after SCA, and extracorporeal CPR. Postcardiac arrest care is considered coronary angiography, which will be performed in an emergency setting. Comatose adult patients with an ROSC after SCA will receive TTM; fever will be actively prevented in these comatose patients after TTM, and other standard critical care interventions will be administered.

### EA treatment

Disposable stainless steel acupuncture needles will be inserted at the Baihui acupoint (GV20, located at the intersection of the sagittal midline and the line linking the ears) and unilaterally on the side of the left leg at the Zusanli acupoint (ST36, situated on the front to one side of the leg, 3 *cun* below the acupoint ST35 and a cross-finger (middle finger) away from the leading edge of the tibia) to a depth of approximately 0.5 cm [28]. Furthermore, an EA instrument (Suzhou Medical Supplies Factory Co., Ltd., Jiangsu, China) will be connected to perform EA stimulation for 30 min using a dense-dispersed wave at frequencies of 20 and 100 Hz. The current intensity will be less than 10 mA (adjusted to induce a slight muscle twitch), and the pulse width will be 0.5 ms. One of the two electrodes of the EA stimulator will be connected to the needle at GV20, and the other electrode will be connected to the needle at ST36. This EA intervention will begin immediately after the ROSC for 20 min and will continue for up to 14 days, discharge, or death, whichever comes first. The EA will be performed by Decai Zhu, Baijian Chen, or Wei Huang, who will conduct the research and have previously received equal training.

### Principles of the formula

GV20 is located at the top of the apex, which is the intersection of three *yang* meridians of the hand, three *yang* meridians of the foot, and the governor vessel. At the same time, the head is the meeting of *yang* and the ancestor of meridian vessels. The functional brain injury after PCAS is located in the head, and the pathogenesis is *yin-yang* disharmony, the reversal of *qi* and blood reversal. Therefore, the choice of acupuncture at GV20, where vessels and *qi* converge, plays an essential role in regulating the body’s *yin-yang* balance [29, 30].

ST36 is the acupoint of the stomach meridian (ST), the *sea point* in the *five transport points*. The stomach is the sea of water and food and the source of *qi* and blood engendering transformation. It has an exterior and interior relationship with the spleen meridian (SP), and the spleen and stomach are the roots of acquisition. “Suwen” indicated that “the treatment of wilting disease takes *yang* brightness meridian alone.” Acupuncture at ST36 can stimulate the *qi* of the viscera and bowels, encourage the movement of qi and blood, and dredge the block of the meridian vessel [31].

EA at GV20 and ST36 can restore *yang* to prevent collapse and open the orifices. According to long-term experience, acupuncture used in our trials is considered safe for patients who suffer from SCA. However, according to modern clinical research principles, it has been criticized for a lack of adequate assessment. Therefore, we designed this observational trial to evaluate the efficacy of EA.

### Outcome measures

The primary feasibility outcomes are as follows:

- Evaluate the acceptability and compliance of EA in PCAS as an additional intervention of usual care.
- Explore the feasibility of recruiting, randomizing, and retaining participants.
- Evaluate outcome measures’ appropriateness.
- Collect data for effect size calculation in future sample size calculating.
- Develop an appropriate protocol for further study.

The length of time for recruitment of 50 eligible patients, recruitment rate and dropout rate will be measured. Successful recruitment is defined as at least half (50%) of eligible patients enrolled, with a dropout rate of no more than 20%.

The secondary patient-centred outcomes include the following:

- We define a favourable neurological outcome as a cerebral performance category (CPC) score of 1 or 2 on the 28th day after the ROSC. We define an ROSC as a spontaneous pulse and blood pressure, an abrupt sustained increase in end-tidal CO_2_ partial pressure (PETCO_2_) (typically ≥ 40 mmHg), or spontaneous arterial pressure waves with intra-arterial monitoring.
- Neurological scores: Glasgow Coma Scale (GCS) score, CPC score, modified Rankin Scale (mRS) score, and cranial computed tomography (CT) findings;
- Evaluation of cardiac function: troponin and lactic acid levels and echocardiography parameters.
- Sequential Organ Failure Assessment (SOFA) scores
- Acute Physiology and Chronic Health Evaluation (APACHE) II scores
- Length of stay in the ICU, length of hospital stay, hospital mortality/discharge, and hospital costs
- Adverse events

### Data acquisition and biological parameter assessment

The recruited patients in this study will receive a participant ID, which will be labelled in the chart containing the personal information of the participants. Only investigators involved in this study will have the right to access the participant IDs on an as-needed basis.

The following data will be collected from all participants:

- Clinical, laboratory, and imaging-study findings on day 0, day 3, day 7, day 14, and day 28 (if available)
- Emergency medical service (EMS) record, ICU mortality, ICU length of stay, hospital mortality, hospital length of stay, 28-day all-cause mortality, and inpatient cost
- Details on the decision-making process

If patients are discharged early, investigators will call them back to the hospital as outpatients for free data acquisition and examinations.

**Fig. 2:**
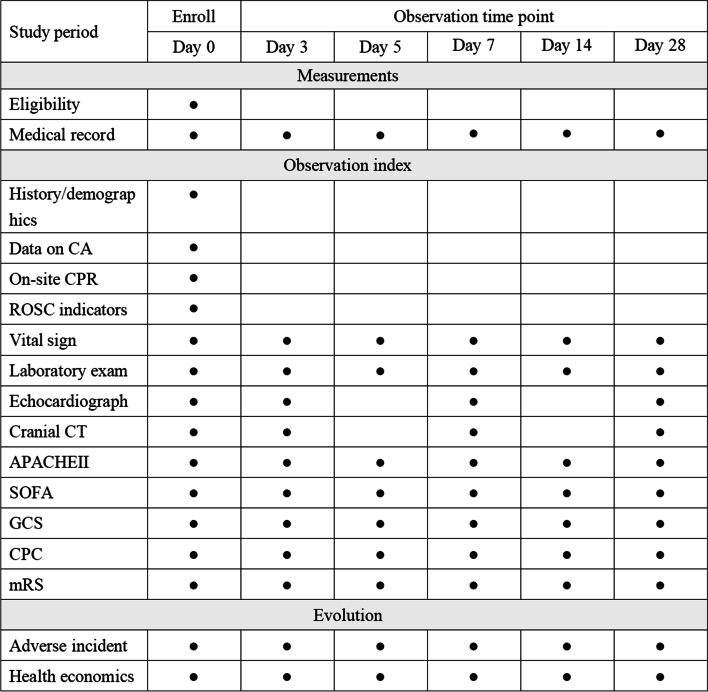
Standard Protocol Items: Recommendations for Interventional Trials (SPIRIT) timeline of measurements. Laboratory examinations include tests for inflammation parameters (C-reactive protein and procalcitonin), complete blood counts, general urine analyses, liver function tests (ALT, AST, ALP, TBIL, and GGT), renal function tests (BUN, Scr), and tests to measure troponin and lactic acid

The clinical parameters will include age, sex, cause of SCA, comorbidities, APACHE II scores, and SOFA scores. The EMS record contains the time of the call for EMS, the time of arrival, the initial cardiac rhythm, and management information. The data above will be collected on the day of enrolment and 28 days after registration. The schedule of the trial is shown in Fig. 2.

Routine laboratory examinations (complete blood counts (CBCs), C-reactive protein (CRP) and procalcitonin (PCT) tests, liver function tests (ALT, AST, ALP, TBIL, and GGT), and renal function tests (BUN and Scr)) will be performed by the clinical laboratory of Guangdong Provincial Hospital of Chinese Medicine.

Investigators will be trained to collect trial data according to the standard protocol. Data will ultimately be input into the Clinical Trial Management Public Platform, ResMan, designed for clinical trials. Double data entry will be performed by two researchers independently and will be checked by the third data clerk separately. All collected forms will be kept in the scientific research department of Guangdong Provincial Hospital of Chinese Medicine. Investigators will be trained to collect trial data according to the standard protocol.

Any adverse event that occurs in participants, regardless of whether it is related to the EA, will be recorded from recruitment to the 28th day. Severe adverse events and unexpected adverse events will be reported to the Ethics Committee within 2 days. EA will be suspended, and symptomatic treatment will be offered when needed. The principal investigator will decide whether the participants should discontinue the trial.

Both data and safety will be monitored by the Institutional Review Board and the scientific research department of Guangdong Provincial Hospital of Chinese Medicine, independent of the sponsor, with no competing interests.

### Statistical analysis

The data will be analysed statistically according to the method of a previous study [32]. Enrolled participants who cannot continue the study or follow up after treatment will remain in an “intention to treat” analysis. All statistical analyses will be performed with SPSS (version 17.0, Chicago, USA) for Windows by researchers not implementing the intervention. The analysis of this trial will be mainly descriptive focusing on estimation rather than hypothesis testing. All baseline outcomes will be summarized for each group using the appropriate descriptive statistics. No formal comparison of groups will take place. A full statistical analysis plan will be developed prior to the final analysis of the trial.

## Discussion

In addition to high mortality, SCA remains a cause of disability in survivors [17]. Hypothermia has been applied for the treatment of SCA from the 1950s to the present. Hypothermia has been shown to improve neurological outcomes and survival in patients with severe ischaemia–reperfusion brain injury after the ROSC [6, 33–35]. Animal studies have shown that the mechanism underlying the effect of mild hypothermia on PCAS is complicated and that it does not merely involve a decline in brain metabolism [36, 37]. Furthermore, TTM is currently the only clinically proven way to improve neurological outcomes and survival after the ROSC. However, TTM is still associated with a series of problems that have not been completely resolved.

Retrospective studies have shown that for IHCA, the earlier TTM is implemented, the higher the proportion of favourable neurological outcomes, and this trend is also seen in the survival rate [38, 39]. Because of the low quality of evidence, the data showing that the effects of prehospital induction of cooling are superior to those of in-hospital initiation of cooling are not convincing. Moreover, prehospital cooling might result in higher rearrest rates after the ROSC [40, 41]. The delay of mild hypothermia in retrospective studies may be related to problems such as a lack of simplified procedures and experience in PCAS, hemodynamic instability, recurrence, and coronary angiography. Therefore, whether early initiation of mild hypothermia can result in a favourable neurological outcome and induce hypothermia remains controversial.

Acupuncture is an integral part of TCM. Many documents show that it was the primary means of treating emergencies such as SCA in ancient times. Whether acupuncture can be used as a new technique in CPR to improve survival with a favourable outcome is worthy of in-depth study.

Studies have shown that after EA, oligodendrocyte regeneration increases [42, 43], motor and memory function improves [44, 45], and cognitive impairment is ameliorated [46]. The role of EA is related to the upregulated expression of neurotrophin-4/5-tyrosine receptor kinase B (NT4/5-TrkB) signalling [42], cAMP response element-binding protein/brain-derived neurotrophic factor (CREB/BDNF) signalling [44, 45, 47, 48], stromal cell-derived factor-1α (SDF-1α) [49], B cell lymphoma-2 (Bcl-2) and B cell lymphoma-2 related X protein (Bax) [46, 50]. Animal studies have shown that acupuncture at GV20 and ST36 can significantly reduce blood–brain barrier permeability and brain oedema, and the effect of acupuncture may inhibit the expression of phosphorylated caveolin-1 in endothelial cells [51]; downregulate reactive oxygen species (ROS) and NADPH oxidase type 4 (NOX4) [52]; and upregulate matrix metalloproteinase-2 (MMP2), aquaporin-4 (AQP4) and aquaporin-9 (AQP9) [53]. It was found in a middle cerebral artery occlusion (MCAO) rat model study that EA could inhibit neuronal apoptosis, impede the activation of microglia and inhibit inflammatory responses [54]. Acupuncture may involve specific effects on the protease A (PKA)/CREB pathway [23] and erythropoietin (EPO)-mediated Janus family tyrosine kinase 2 (JAK2) signal transduction and transcription activator 3 (STAT3) cell pathway [22], reduce S100B-mediated neurotoxicity [55] and finally achieve anti-apoptosis. In addition, anti-ischaemic apoptosis may be obtained through the downregulation of the oxidative stress response, which is associated with a decrease in tumour necrosis factor α (TNF-α), interleukin 6 (IL-6), neuron-specific enolase (NSE), malondialdehyde (MDA), superoxide dismutase (SOD), and catalase (CAT) in the serum and hippocampus [56]. Furthermore, EA reduced the infarct area and increased the ejection fraction (EF) by inhibiting the expression of NLR family pyrin domain containing 3 (NLRP3) and AMP-activated protein kinase (AMPK)-dependent autophagy in an animal study of an acute myocardial infarction rat model [57, 58].

Our decision to use an EA in PCAS is based on the results of our retrospective study published in Chinese, in which we found that EA attenuates the multiorgan failure of patients with PCAS [59], and those of a systematic review indicating that acupuncture ameliorates the injuries induced by experimental sepsis [60]. Whether EA is associated with mortality or a favourable neurological outcome after PACS is unclear. However, this trial is the first registered interventional and experimental study evaluating the potential benefits of EA on the neurological outcome of PCAS. The results of this trial might establish the importance of EA as a promising complementary strategy for the treatment of PCAS.

This study is the first study of EA for the treatment of SCA. The initial design was to consider SCA due to all causes; however, the ethics committee suggested that a pilot study in a focused area was needed to clarify the feasibility and safety of EA. The next step will be to expand the research. Therefore, there are several limitations to this trial. First, this is a pilot study with a small sample size and no blinding of the recruited participants. Second, the causes of SCA vary, and EA may be useful only for some causes of SCA, which could ultimately lead to bias; therefore, matched control and subgroup analyses are needed. Third, the treatment strategy received by ICU patients is highly complicated; therefore, the results of this trial might not be adequately explained solely by the therapeutic effect of EA. Fourth, as stated throughout this paper, this was a pilot study and no sample size calculation was performed; this was not powered for hypothesis testing of clinical outcomes. The clinical results will be treated as preliminary and interpreted with caution.

### Trial status

We are currently recruiting participants.

## Supplementary Information

**Additional file 1.** SPIRIT 2013 Checklist.

## Data Availability

The ethical, consent, and funding documents of this study are available for review by the Editorial Office. Ruifeng Zeng and Banghan Ding are responsible for the data and will have the final dataset. Trial results will be reported to the Guangzhou Science and Technology Department for public publication. Additionally, the investigators plan to publish the results in journals. Participants can reach out for information on the study progress and related data.

## Abbreviations

SCA: Sudden cardiac arrest
ROSC: Return of spontaneous circulation
MODS: Multiple organ dysfunction syndrome
PCAS: Postcardiac arrest syndrome
TTM: Targeted temperature management
TCM: Traditional Chinese medicine
CPR: Cardiopulmonary resuscitation
ACLS: Advanced cardiovascular life support
OHCA: Out-of-hospital cardiac arrest
IHCA: In-hospital cardiac arrest
ICU: Intensive care unit
CCF: Chest compression fraction
EA: Electroacupuncture
BLS: Basic life support
CPC: Cerebral performance category
GCS: Glasgow Coma Scale
mRS: Modified Rankin scale
CT: Computed tomography
SOFA: Sequential Organ Failure Assessment
APACHE: Acute Physiology and Chronic Health Evaluation
EMS: Emergency medical service
CBC: Complete blood count
CRP: C-reactive protein
PCT: Procalcitonin
NT4/5-TrkB: Neurotrophin-4/5-tyrosine receptor kinase B
CREB: cAMP response element binding protein
BDNF: Brain-derived neurotrophic factor
SDF-1α: Stromal cell-derived factor-1α
Bcl-2: B cell lymphoma-2
Bax: B cell lymphoma-2-related X protein
ROS: Reactive oxygen species
NOX4: NADPH oxidase type 4
MMP2: Matrix metalloproteinase-2
AQP: Aquaporin
MCAO: Middle cerebral artery occlusion
PKA: Protease A
EPO: Erythropoietin
JAK2: Janus family tyrosine kinase 2
STAT3: Signal transduction and transcription activator 3
TNF-α: Tumor necrosis factor α
IL-6: Interleukin 6
NSE: Neuron-specific enolase
MDA: Malondialdehyde
SOD: Superoxide dismutase
CAT: Catalase
EF: Ejection fraction
NLRP3: NLR family pyrin domain containing 3
AMPK: AMP-activated protein kinase
SPIRIT: Standard Protocol Items: Recommendations for Interventional Trials
