# Supplementary material for: Feasibility of electroacupuncture at Baihui (GV20) and Zusanli (ST36) on survival with a favorable neurological outcome in patients with postcardiac arrest syndrome after in-hospital cardiac arrest: study protocol for a pilot randomized controlled trial
Source: Pilot Feasibility Stud. 2023 Jan 13;9:8. doi: 10.1186/s40814-023-01239-9 (PMC9837931; doi:10.1186/s40814-023-01239-9)
Supplement: Supplementary file 1 — Additional file 1. SPIRIT 2013 Checklist. [file 40814_2023_1239_MOESM1_ESM.docx]

**Additional file 1:SPIRIT Checklist: Recommended Items to Address in a Clinical Trial Protocol and Related Documents**

| **Section/Item**  **Administrative information** | **Item Number** | **Description** |
| --- | --- | --- |
| Title | 1 | Descriptive title identifying the study design, population, interventions, and, if applicable, trial acronym  Effect of Electroacupuncture at Baihui (GV20) and Zusanli (ST36) on survival with a favorable neurological outcome of Post-cardiac Arrest Syndrome: study protocol for a pilot randomized controlled trial |
| Trial registration | 2a | Trial identifier and registry name. If not yet registered, name of intended registry.  Trial identifier：chictr.org.cn: ChiCTR2000040040  Registry name: Effect of Electroacupuncture on survival with a favorable neurological outcome of Post-cardiac Arrest Syndrome: a pilot randomized controlled trial |
|  | 2b | All items from the World Health Organization Trial Registration Data Set (Appendix Table, available at [www.annals.org](http://www.annals.org))  Not applicable for this trial. |
| Protocol version | 3 | Date and version identifier  Date December 5, 2020 and version 1.0 |
| Funding | 4 | Sources and types of financial, material, and other support  This work was supported by the grant of Project of Guangzhou Science and Technology Department, China (201803010030), Guangdong Science and Technology Projects, China (2017ZC0164), and Guangdong Provincial Key Laboratory of Research on Emergency in TCM, China (2017B030314176). |
| Roles and responsibilities | 5a | Names, affiliations, and roles of protocol contributors  Ruifeng Zeng^1,2,3^, Fang Lai^1,4^，Decai Zhu^1,4^, Baijian Chen^1,2^, Lanting Tao^1,5^, Wei Huang^1,2^, Manhua Huang^1,2^, Chengzhi Lai^5^, Banghan Ding^1,2*^  ^1^ The Second Affiliated Hospital of Guangzhou University of Chinese Medicine, Guangdong Provincial Key Laboratory of Research on Emergency in TCM, Guangzhou 510120, Guangdong, China.  ^2^ Guangdong Provincial Hospital of Chinese Medicine, Guangzhou 510120, Guangdong, China.  ^4^ Fangcun Branch Hospital of Guangdong Provincial Hospital of Chinese Medicine, Guangzhou 510145, Guangdong, China.  ^5^ Ersha Branch Hospital of Guangdong Provincial Hospital of Chinese Medicine, Guangzhou 510105, Guangdong, China.  Authors’ contributions: Ruifeng Zeng and Banghan Ding drafted this manuscript. Ruifeng Zeng, Fang Lai, and Banghan Ding designed the described study. Decai Zhu, Baijian Chen, Lanting Tao, and Wei Huang conduct the research. Chengzhi Lai and Manhua Huang acquire data. Ruifeng Zeng and Chengzhi Lai made statistical analysis. Chenzhi Lai and Lanting Tao monitor the process of trial implementation. And all authors read and approved this final manuscript. |
|  | 5b | Name and contact information for the trial sponsor  Guangzhou Science and Technology Department, 47 Lianxin Road, Yuexiu District, Guangzhou 510033, China  Guangdong Science and Technology Department, 171 Lianxin Road, Yuexiu District, Guangzhou 510033, China  Guangdong Provincial Key Laboratory of Research on Emergency in TCM, 111 Dade Road, Yuexiu District, Guangzhou 510120, China |
|  | 5c | Role of study sponsor and funders, if any, in study design; collection, management, analysis, and interpretation of data; writing of the report; and the decision to submit the report for publication, including whether they will have ultimate authority over any of these activities  Funders of this study have no role in any abovementioned activities. |
|  | 5d | Composition, roles, and responsibilities of the coordinating center, steering committee, end point adjudication committee, data management team, and other individuals or groups overseeing the trial, if applicable (see item 21a for DMC)  Ruifeng Zeng and Banghan Ding drafted this manuscript. Ruifeng Zeng, Fang Lai, and Banghan Ding designed the described study. Decai Zhu, Baijian Chen, Lanting Tao, and Wei Huang conduct the research. Chengzhi Lai and Manhua Huang acquire data. Ruifeng Zeng and Chengzhi Lai made statistical analysis. Chengzhi Lai and Lanting Tao monitor the process of trial implementation. The investigation is conducted in accordance with national laws and the Declaration of Helsinki principles and received oversees from Guangdong Provincial Hospital of Chinese Medicine Institutional Review Board and Scientific Research Department of Guangdong Provincial Hospital of Chinese Medicine. |
| **Introduction** | | |
| Background and rationale | 6a | Description of research question and justification for undertaking the trial, including summary of relevant studies (published and unpublished) examining benefits and harms for each intervention  At present, even the first-line medication, epinephrine, still had no evidence to prove the favorable neurologic outcome in cardiac arrest (CA). The high mortality of post-cardiac arrest syndrome (PCAS) can be attributed to brain injury, myocardial dysfunction, systemic ischemia/reperfusion response, and persistent precipitating pathology. And target temperature management, the only clinically proven method in PCAS, still has a series of problems unclear. Acupuncture is one crucial therapy in traditional Chinese medicine. We suppose electroacupuncture (EA) might have therapeutic benefits in PCAS based on the previous studies. This study is to evaluate the effect of EA on PCAS patients. |
|  | 6b | Explanation for choice of comparators  We choose blank as the comparator in this study. |
| Objectives | 7 | Specific objectives or hypotheses  The purpose of this study is to evaluate whether EA can improve survival with a favorable neurological outcome of PCAS. |
| Trial design | 8 | Description of trial design, including type of trial (e.g., parallel group, crossover, factorial, single group), allocation ratio, and framework (e.g., superiority, equivalence, noninferiority, exploratory)  This study is a prospective pilot randomized controlled clinical trial carried out in the Emergency Department of Guangdong Provincial Hospital of Chinese Medicine and Intensive Care Unit of Fangcun Branch Hospital of Guangdong Provincial Hospital of Chinese Medicine, embedded with a study of survival with a favorable neurological outcome evaluation of PCAS. |
| **Methods** | | |
| Participants, interventions, and outcomes | | |
| Study setting | 9 | Description of study settings (e.g., community clinic, academic hospital) and list of countries where data will be collected. Reference to where list of study sites can be obtained  This multi-center randomized controlled clinical trial will be carried out in the Emergency Department, Guangdong Provincial Hospital of Chinese Medicine (located at No.111 Dade Road, Guangzhou 510120) and intensive care unit, Fangcun Branch Hospital of Guangdong Provincial Hospital of Chinese Medicine (located at No.36 Yongan Street, Guangzhou 510145). |
| Eligibility criteria | 10 | Inclusion and exclusion criteria for participants. If applicable, eligibility criteria for study centers and individuals who will perform the interventions (e.g., surgeons, psychotherapists)  Inclusion criteria are:  1. Cardiac arrest is caused by respiratory failure or hypovolemic shock;  2. In-Hospital Cardiac Arrest;  3. Aged 18-85 years old;  4. Patients with return of spontaneous circulation (ROSC) that is sustained for ≥20 minutes;  5.Glasgow coma score ≤8 after ROSC, and before sedation (if any) ;  6.The legal representative signed the informed consent;  7.advanced cardiovascular life support is conducted.  Exclusion criteria are:  1. No-flow time >10 min (time from collapse to initiation of external cardiac massage);  2. Low-flow time >60 min (time from initiation of external cardiac massage to ROSC);  3. Major hemodynamic instability (defined as a continuous epinephrine or norepinephrine infusion at a flow rate >1 μg/Kg/min);  4. Cardiac arrest caused by advanced tumor and other end-of-state diseases;  5. Cardiac arrest caused by an irreversible cause (such as Severe trauma and poisoning);  6. Cardiogenic cardiac arrest (acute infarction, malignant arrhythmia, heart failure, etc.);  7. Acupoint with lesions, wounds, or skin diseases affects acupuncture;  8. Allergic persons, or known to be allergic to treatment, such as metal;  9. pregnant or lactating women. |
| Interventions | 11a | Interventions for each group with sufficient detail to allow replication, including how and when they will be administered  Consenting eligible patients will receive EA, other than which participants will receive standard therapy according to American Heart Association guidelines for CPR recommendations, while another adjunctive treatment is prohibited.  Standard therapy is based on current recommendations13, 24-26, including basic life support (BLS), advanced cardiovascular life support (ACLS), and post-cardiac arrest care. BLS includes immediate recognition of CA, activation of the EMS, early CPR, and rapid defibrillation. ACLS is consists of airway control and ventilation, antiarrhythmic drugs and vasopressors during and immediately after CA, and extracorporeal CPR. Post-cardiac arrest care is considered coronary angiography. It should be performed emergently, comatose adult patients with ROSC after CA have TTM, actively prevent fever in comatose patients after TTM, and other standard critical care interventions.  EA treatment involve the disposable stainless needles acupuncture at the Baihui acupoint (GV20, located at the intersection of sagittal midline and the line linking two ears), and insert unilaterally on the left-leg side at Zusanli acupoints (ST36, situated in front to one side of the leg, 3 cun below the point ST35, away from the tibia leading edge of a cross-fingers (middle finger)), to a depth of approximately 0.5cm27. Furthermore, an EA instrument (Suzhou Medical Supplies Factory Co., Ltd.; Jiangsu, China) is connected to perform EA stimulation for 30 min, using a dense-dispersed wave at frequencies of 20 and 100 Hz. The current intensity of less than 10mA (adjusted to induce a slight twitch of the muscle) and the pulse width is 0.5ms. One of the two electrodes of EA stimulator is connected to the needle at GV20, and the other electrode is connected ST36. This EA intervention will be continued up to 14 days, ICU discharge, or death, whichever came first. |
|  | 11b | Criteria for discontinuing or modifying allocated interventions for a given trial participant (e.g., drug dose change in response to harms, participant request, or improving/worsening disease)  Participants are allowed to withdraw from the trial for any reason at any time. Researchers can remove participants from the trial to ensure their safety or maintain the quality of the trial. Participants with any of the following conditions may be removed from the study: 1) The legal representative of the patient requested to withdraw from the study; 2) death. |
|  | 11c | Strategies to improve adherence to intervention protocols, and any procedures for monitoring adherence (e.g., drug tablet return, laboratory tests)  Investigators experimenting will be trained before the start. Chengzhi Lai and Lanting Tao, who are not involved in participants’ enrollment and data analysis, monitor the implementation at least once per month in the process of trial. |
|  | 11d | Relevant concomitant care and interventions that are permitted or prohibited during the trial  Concomitant medications as the American Heart Association guidelines for CPR recommended are permitted, another adjunctive therapy is prohibited. |
| Outcomes | 12 | Primary, secondary, and other outcomes, including the specific measurement variable (e.g., systolic blood pressure), analysis metric (e.g., change from baseline, final value, time to event), method of aggregation (e.g., median, proportion), and time point for each outcome. Explanation of the clinical relevance of chosen efficacy and harm outcomes is strongly recommended  The primary endpoints are as follows:   - Evaluate the acceptability and compliance of EA in PCAS as an additional intervention of usual care. - Explore the feasibility of recruiting, randomizing and retaining participants. - Evaluate outcome measures’ appropriateness. - Collect data for effect size calculation in future sample size calculating. - Develop an appropriate protocol for further study.   The length of time for recruitment of 50 eligible patients, recruitment rate and dropout rate will be measured. Successful recruitment is defined as at least half (50%) of eligible patients enrolled, with a dropout rate of no more than 20%.  The secondary endpoints include the following:   - We define a favourable neurological outcome as a cerebral performance category (CPC) score of 1 or 2 on the 28^th^ day after the ROSC. We define an ROSC as a spontaneous pulse and blood pressure, an abrupt sustained increase in end-tidal CO_2_ partial pressure (PETCO_2_) (typically ≥40 mmHg), or spontaneous arterial pressure waves with intra-arterial monitoring. - Neurological scores: Glasgow Coma Scale (GCS) score, CPC score, modified Rankin Scale (mRS) score, and cranial computed tomography (CT) findings; - Evaluation of cardiac function: troponin and lactic acid levels and echocardiography parameters. - Sequential Organ Failure Assessment (SOFA) scores. - Acute Physiology and Chronic Health Evaluation (APACHE) II scores. - Length of stay in the ICU, length of hospital stay, hospital mortality/discharge, and hospital costs. |
| Participant timeline | 13 | Time schedule of enrollment, interventions (including any runins and washouts), assessments, and visits for participants. A schematic diagram is highly recommended (Figure).  SPIRIT timeline of measurements is listed in Figure 2. |
| Sample size | 14 | Estimated number of participants needed to achieve study objectives and how it was determined, including clinical and statistical assumptions supporting any sample size calculations  Since there is no previous clinical study on EA for the survival of PCAS and in view of this trial’s pilot nature, we will take the recommendation of 20 participants or more to achieve sufficient precision for subsequent studies’ sample size calculation. We plan to recruit a total of 50 participants since a 20% dropout rate is expected. |
| Recruitment | 15 | Strategies for achieving adequate participant enrollment to reach target sample size  Close collaboration between physicians and researchers in Emergency Departments and Intensive Care Unit Departments. At least one researcher of the study group will be noticed with every potential eligible participant to initiate the standard recruitment protocol. |
| Assignment of interventions (for controlled trials) | | |
| Allocation Sequence generation | 16a | Method of generating the allocation sequence (e.g., computer-generated random numbers), and list of any factors for stratification. To reduce predictability of a random sequence, details of any planned restriction (e.g., blocking) should be provided in a separate document that is unavailable to those who enroll participants or assign interventions.  Eligible patients enrolled in this trial are being randomly assigned to either the EA group or the control group as a 1:1 ratio. Randomization assignment will be carried out by a researcher (Fang Lai) not involved in the treatment and assessment. This randomization is run with a block randomization size of four by SPSS 17.0. |
| Allocation concealment mechanism | 16b | Mechanism of implementing the allocation sequence (e.g., central telephone; sequentially numbered, opaque, sealed envelopes), describing any steps to conceal the sequence until interventions are assigned  Allocation will be sequentially numbered stored in sealed envelopes until interventions assigned. |
| Implementation | 16c | Who will generate the allocation sequence, who will enroll participants, and who will assign participants to interventions  Fang Lai will generate the allocation sequence, Decai Zhu and Baijian Chen will enroll participants and assign participants to interventions according to pre-prepared allocation sequence. |
| Blinding (masking) | 17a | Who will be blinded after assignment to interventions (e.g., trial participants, care providers, outcome assessors, data analysts), and how  The laboratory technicians, radiologists, and statisticians will be blinded to the treatments, with no unblinding needed under any condition. |
|  | 17b | If blinded, circumstances under which unblinding is permissible, and procedure for revealing a participant’s allocated intervention during the trial  There is no blinding for treating physicians or participants in this trial, only the laboratory technicians and the biostatisticians responsible for the statistical analysis will be blinded to the assigned treatments. Therefore, no unblinding is needed under any circumstance. |
| Data collection, management, and analysis | | |
| Data collection methods | 18a | Plans for assessment and collection of outcome, baseline, and other trial data, including any related processes to promote data quality (e.g., duplicate measurements, training of assessors) and a description of study instruments (e.g., questionnaires, laboratory tests) along with their reliability and validity, if known. Reference to where data collection forms can be found, if not in the protocol.  Investigators are trained to collect trial data according to the standard protocol. Data will ultimately be input into the Clinical Trial Management Public Platform, ResMan, designed for the clinical trials. All collected forms will be kept in the scientific research department of Guangdong provincial hospital of Chinese Medicine.Investigators are trained to collect trial data according to the standard protocol. |
|  | 18b | Plans to promote participant retention and complete follow-up, including list of any outcome data to be collected for participants who discontinue or deviate from intervention protocols  Follow-up phone call will be conducted to complete follow-up. |
| Data management | 19 | Plans for data entry, coding, security, and storage, including any related processes to promote data quality (e.g., double data entry; range checks for data values). Reference to where details of data management procedures can be found, if not in the protocol.  Double Data entry will be done by two researchers independently and be checked by the third data clerk separately. |
| Statistical methods | 20a | Statistical methods for analyzing primary and secondary outcomes. Reference to where other details of the statistical analysis plan can be found, if not in the protocol.  The data will be analyzed statistically according to a previous study^32^. Quantitative numerical results with normal distribution are presented as mean±SD, while non-normal distribution ones are presented as median and interquartile range. Categorical variables are expressed as frequencies or numbers. Levene's test assesses the equality of variances between groups. Unpaired Student’s t-test is used for normally distributed quantitative variables, while the Wilcoxon nonparametric statistic is used for non-normally distributed ones. Pearson's chi-square test or Fisher exact test is used for categorical data when appropriate. Enrolled subjects that are not able to continue the study or follow up after treatment will remain to perform an “intention to treat” analysis. A probability value of P＜0.05 will be considered statistically significant. All statistical analyses will be performed with SPSS (version 17.0, Chicago, USA) for Windows by researchers not implementing the trial. |
|  | 20b | Methods for any additional analyses (e.g., subgroup and adjusted analyses)  Not applicable for this trial. |
|  | 20c | Definition of analysis population relating to protocol nonadherence (e.g., as-randomized analysis), and any statistical methods to handle missing data (e.g., multiple imputation)  Enrolled subjects that are not able to continue the study or follow up after treatment will remain to perform an “intention to treat” analysis. |
| Monitoring | | |
| Data monitoring | 21a | Composition of DMC; summary of its role and reporting structure; statement of whether it is independent from the sponsor and competing interests; and reference to where further details about its charter can be found, if not in the protocol. Alternatively, an explanation of why a DMC is not needed.  Data and safety will be monitored by Guangdong Provincial Hospital of Chinese Medicine Institutional Review Board and Scientific Research Department of Guangdong Provincial Hospital of Chinese Medicine, which are independent from the sponsor with no competing interests. |
|  | 21b | Description of any interim analyses and stopping guidelines, including who will have access to these interim results and make the final decision to terminate the trial  Not applicable for this trial. |
| Harms | 22 | Plans for collecting, assessing, reporting, and managing solicited and spontaneously reported adverse events and other unintended effects of trial interventions or trial conduct  Any adverse event that occurred to participants, whether related to the EA, will be recorded, from recruitment to the 28th day. Severe adverse events and unexpected adverse events will be reported to the Ethics Committee within 2 days. The EA will be suspended, and symptomatic treatment will be offered when needed. The principal investigator will decide whether the participants should discontinue the trial. |
| Auditing | 23 | Frequency and procedures for auditing trial conduct, if any, and whether the process will be independent from investigators and the sponsor  Chengzhi Lai and Lanting Tao, who are not involved in participants’ enrollment and data analyzed, monitor the implementation at least once per month in the process of trial. |
| **Ethics and dissemination** | | |
| Research ethics approval | 24 | Plans for seeking REC/IRB approval  Ethics approved from the Guangdong Provincial Hospital of Chinses Medicine Institutional Review Board (approval number ZF2020-051-01). |
| Protocol amendments | 25 | Plans for communicating important protocol modifications (e.g., changes to eligibility criteria, outcomes, analyses) to relevant parties (e.g., investigators, RECs/IRBs, trial participants, trial registries, journals, regulators)  If there are important protocol changes, a new version of protocol will be submitted to Guangdong Provincial Hospital of Chinses Medicine Institutional Review Board. |
| Consent or assent | 26a | Who will obtain informed consent or assent from potential trial participants or authorized surrogates, and how (see item 32)  Decai Zhu, Baijian Chen, and Wei Huang will obtain informed consent from potential trial participants. |
|  | 26b | Additional consent provisions for collection and use of participant data and biological specimens in ancillary studies, if applicable  Not applicable for this trial. |
| Confidentiality | 27 | How personal information about potential and enrolled participants will be collected, shared, and maintained in order to protect confidentiality before, during, and after the trial  The patient recruited will receive a participation ID in this study, with which personal information of participants is labeled in the chart. Only investigators involved in this study have the right to access to participants' ID identity on an as-needed basis. |
| Declaration of interests | 28 | Financial and other competing interests for principal investigators for the overall trial and each study site  None. |
| Access to data | 29 | Statement of who will have access to the final trial data set, and disclosure of contractual agreements that limit such access for investigators  Ruifeng Zeng and Banghan Ding are responsible for the data and have the final dataset. |
| Ancillary and post-trial care | 30 | Provisions, if any, for ancillary and post-trial care, and for compensation to those who suffer harm from trial participation  Not applicable. |
| Dissemination policy | 31a | Plans for investigators and sponsor to communicate trial results to participants, health care professionals, the public, and other relevant groups (e.g., via publication, reporting in results databases, or other data-sharing arrangements), including any publication restrictions  Trial results will be reported to Guangdong Science and Technology Department to publish to the public. Also, the investigators plan to publish the results in journals. Participants can reach out for information of the study progress and any related information. |
|  | 31b | Authorship eligibility guidelines and any intended use of professional writers  We haven’t used such a service. |
|  | 31c | Plans, if any, for granting public access to the full protocol, participant-level data set, and statistical code  This was already mentioned in 18a and 31a. |
| **Appendices** | | |
| Informed consent materials | 32 | Model consent form and other related documentation given to participants and authorized surrogates  Model consent form and other related documentation given to participants and authorized surrogates are available from the corresponding author on reasonable request. |
| Biological specimens | 33 | Plans for collection, laboratory evaluation, and storage of biological specimens for genetic or molecular analysis in the current trial and for future use in ancillary studies, if applicable  Blood samples from participants will be collected and stored at -80℃ for the final analysis. |
